# Effects of Anterior Capsulotomy on Decision Making in Patients with Refractory Obsessive–Compulsive Disorder

**DOI:** 10.3389/fpsyg.2017.01814

**Published:** 2017-10-17

**Authors:** Chencheng Zhang, Yilin Chen, Shuaiwei Tian, Tao Wang, Yile Xie, Haiyan Jin, Guozhen Lin, Hengfen Gong, Kristina Zeljic, Bomin Sun, Tianming Yang, Shikun Zhan

**Affiliations:** ^1^Department of Functional Neurosurgery, Ruijin Hospital, Shanghai Jiao Tong University School of Medicine, Shanghai, China; ^2^Institute of Neuroscience, Key Laboratory of Primate Neurobiology, CAS Center for Excellence in Brain Science and Intelligence Technology, Chinese Academy of Sciences, Shanghai, China; ^3^University of Chinese Academy of Sciences, Beijing, China; ^4^Shanghai Jiao Tong University School of Medicine, Shanghai, China; ^5^Department of Psychiatry, Ruijin Hospital, Shanghai Jiao Tong University School of Medicine, Shanghai, China; ^6^Department of Psychiatry, Pudong District Mental Health Center, Shanghai, China

**Keywords:** obsessive–compulsive disorder, anterior capsulotomy, decision-making, Iowa gambling task

## Abstract

Despite various lines of evidence implicating impaired decision-making ability in individuals with obsessive–compulsive disorder (OCD), neuropsychological investigation has generated inconsistent findings. Although the cortico-striato-thalamo-cortical (CSTC) circuitry has been suggested, the involvement of the cortex has not yet been fully demonstrated. Moreover, it is unknown whether surgical intervention on the CSTC circuitry results in a predicted improvement of decision-making ability of OCD. Here we present a study of decision making based on the Iowa Gambling Task (IGT) to investigate decision making in a large sample of individuals with treatment-resistant OCD with and without anterior capsulotomy (AC). Task performance was evaluated in healthy subjects, individuals with OCD that had not undergone surgery, and postsurgical OCD patients with AC. The latter group was further divided into a short-term postsurgical group and a long-term postsurgical group. We found that the OCD patients without surgery performed significantly worse than the healthy controls on the IGT. There were no significant differences in decision-making between the presurgical OCD patients and those at the short-term postsurgical follow-up. Decision-making ability of the long-term postsurgical OCD patients was improved to the level comparable to that of healthy controls. All clinical symptoms (OCD, depression, and anxiety) assessed by psychiatric rating scales were significantly alleviated post-surgically, but exhibited no correlation with their IGT task performance. Our findings provide strong evidence that OCD is linked to impairments in decision-making ability; that impaired CSTC circuitry function is directly involved in the manifestation of OCD; and that AC related improvements in cognitive functions are caused by long-term plasticity in the brain circuitry.

## Introduction

Obsessive–compulsive disorder (OCD) is a symptomatologically heterogeneous psychiatric disorder with a worldwide lifetime prevalence ranging from 2 to 3% that often leads to chronic disability (Abramowitz et al., 2009). The primary clinical manifestations of this disorder include recurrent, ruminative and anxiety-inducing thoughts (obsessions) and/or repetitively ritualized mental acts or behaviors (compulsions). Existing treatment methods entailing pharmacotherapy and cognitive behavioral therapy (CBT) generally only result in partial symptom improvements, with roughly one third of cases continuing to exhibit severe chronic illness that is resistant to treatment (Bloch et al., 2006, 2012; Mallet et al., 2008; Greenberg et al., 2010; Grant, 2014; Odlaug et al., 2014). Neurosurgical intervention has demonstrated substantial symptom alleviation in such cases (Jung et al., 2014). It typically involves either an ablative procedure or reversible neuromodulation using deep brain stimulation (DBS) at locations within the cortico-striato-thalamo-cortical (CSTC) circuitry, from which effects spread throughout the brain (Greenberg et al., 2010; Jung et al., 2014; Pepper et al., 2015).

The CSTC model of OCD is currently the prevailing model in terms of the neural and pathophysiological underpinnings of the disorder (Pauls et al., 2014), and is supported by decades of animal research that have led to findings of a strong association between OCD-like behavior and aberrant functioning of fronto-striatal neural circuitry (Fineberg et al., 2011). The functionally distinct loops comprising this circuitry originate in specific territories of the frontal cortex, project to target areas in the striatum and pass through the basal ganglia to the thalamus, with recurrent projections back to their corresponding frontal cortical regions of origin (Milad and Rauch, 2012; Nakao et al., 2014). Research in humans has been heavily informed by findings in animals, and brain imaging studies have been extensively employed in the investigation and refinement of the CSTC model (Whiteside et al., 2004; Norman et al., 2016). Although this area of research has yielded variable results in terms of specific regions and the direction of dysfunction (increased vs. decreased activity) in different studies, abnormal activity in the fronto-striatal regions is consistently reported. Resting-state functional magnetic resonance imaging (fMRI) studies most often find increased activity in the orbitofrontal cortex (OFC) and striatum, while symptom provocation paradigms most often find increases in the OFC, caudate and anterior cingulate cortex (ACC) (Saxena et al., 2001; Schlösser et al., 2010; Nakao et al., 2014).

Given the aberrant functioning of the OFC in OCD, it is unsurprising that diminished performance on decision-making tasks is commonly reported in affected individuals. Decision-making ability is a multifaceted higher order function with significant real-world implications that relies on intact functioning of the prefrontal cortex for representing and integrating relevant information. The Iowa Gambling Task (IGT), an extensively used decision-making task paradigm, requires the participant to track the stimulus-reward association by distinguishing between the stimuli with an immediate large reward but a long-term negative consequence and the stimuli with an immediate small reward but a long-term positive consequence. Compared to similar neuropsychological tasks, such as the Wisconsin Card Sorting Test (WCST), the IGT is sensitive to damage and/or dysfunction across a wider range of brain areas, including anterior and posterior regions of the orbitofrontal and ventromedial prefrontal cortex, as well as the dorsolateral prefrontal cortex (Bechara et al., 1998, 2001).

Although a number of studies have investigated the relationship between OCD and decision-making ability using IGT, their findings have not been consistent. Cavedini et al. (2002) demonstrated that subjects with OCD performed more poorly on the IGT than subjects with panic disorder and healthy controls (Cavedini et al., 2002). Furthermore, they found poor neuropsychological task performance to be a predictor of poor outcome to pharmacological treatments. Cavallaro et al. (2003) also found that OCD patients performed significantly worse on the IGT than both control subjects and schizophrenia patients (Cavallaro et al., 2003). However, Nielen et al. (2002) and Lawrence et al. (2006) found no differences between the performance of OCD patients and control subjects in the IGT (Nielen et al., 2002; Lawrence et al., 2006). Interestingly, Zhang et al. (2015) recently found that medicated, non-medicated and remitted OCD patients all performed significantly worse on the IGT than comparison subjects (Zhang et al., 2015)^.^ These inconsistencies may in part be due to the heterogeneity of OCD, the methodological differences, and the sampling constraints in some of the studies.

These inconsistent results indicate that the links between OCD, decision-making and altered prefrontal functions remain unclear. Therefore, studies investigating the effects of surgical intervention in the brain circuitry on OCD and decision-making could shed light on the puzzle (Brown et al., 2016). Surprisingly, there has been only one study to date investigating the effects of surgical intervention in OCD on decision-making as assessed by the IGT (Csigo et al., 2010). Csigo and colleagues monitored five refractory OCD patients that underwent anterior capsulotomy (AC), and five non-operated refractory OCD patients over a 2-year period. Their results indicated improvements in IGT performance in the former group at the 1- and 2-year follow-ups (but not at the 1-month or 6-month follow-ups) with no improvements in the non-operated group. Although these results suggest that AC may lead to improved decision-making in OCD, the study was done with a very limited sample size. It is important to understand the difference between short-term and long-term AC effects on decision making, because it would help us to interpret the circuitry affected by AC.

We therefore aimed to conduct a broad and reliable assessment of decision making under risk as measured by the IGT before and after AC at different time points in a large sample of OCD patients as compared to healthy individuals, while also examining the relationship between task performance and scores on a range of clinical measures. We found that significant improvements in the IGT performance after AC happened only in the patient group that had received the surgery for more than 1 year, suggesting a long-term plasticity in the brain circuitry was responsible for such improvements. Furthermore, the improvements in IGT performance could not be explained by their psychiatrical rating scale scores, which showed improvements shortly after AC.

## Materials and Methods

### Participants

The sample consisted of 138 subjects divided into four groups: 31 healthy subjects (HCs; mean age = 37.77, *SD* = 10.83; mean education = 13.30 years, *SD* = 4.61; Female: 14); 51 pre-operative OCD patients (preO; mean age = 30.71, *SD* = 7.62; mean education = 13.07 years, *SD* = 2.58 years; mean illness duration = 10.66 years, *SD* = 7.12 years; Female: 15); 24 post-operative short term OCD patients (postST), 3.00 to 5.25 months (average 4 months) after surgery (mean age = 29.29, *SD* = 5.72; mean education = 13.40 years, *SD* = 1.98 years; mean illness duration = 8.92 years, *SD* = 3.55 years; Female: 8); and 32 post-operative long term OCD patients (postLT), 2.00 to 5.00 years (average 3.00 years) after surgery (mean age = 33.41, *SD* = 7.86; mean education = 13.3 years, *SD* = 2.47 years; mean illness duration = 10.50 years, *SD* = 4.85 years; Female: 12) (**Table 1**). The patients were recruited via the Department of Psychiatry and Department of Functional Neurosurgery at Ruijin Hospital affiliated to Shanghai Jiao Tong University School of Medicine. The three patient groups were recruited independently. The post-operation groups comprised patients visiting the hospital for the post-surgical follow-up. Measurement of illness duration includes the time period until the post-operative follow-up. The Department of Neurosurgery at Ruijin hospital has a well-established long-term treatment program for refractory OCD (Liu et al., 2008; Zuo et al., 2013; Zhan et al., 2014). All study procedures were performed in accordance with the relevant guidelines and regulations (Nuttin et al., 2014). The diagnosis, symptom severity, and refractory disease status of each referred patient was independently confirmed by three licensed psychiatric professionals according to the Mini-International Neuropsychiatric Interview for DSM-IV-TR (M.I.N.I. 6.0.0) after a complete review of their medical history (Sheehan et al., 1998). Patients with a concurrent diagnosis of bipolar disorder or a history of manic/hypomanic episodes, psychotic symptoms, substance abuse, neurological illness or brain injury/trauma were excluded from the study.

**Table 1:** Age and education of the four subject groups.

	Healthy Controls	Pre-operative	Post-operative short term	Post-operative long term	Welch ANOVA	
	Mean (*SD*)	Mean (*SD*)	Mean (*SD*)	Mean (*SD*)	*F^∗^*	*P*
Age (years)	37.77 (10.83)	30.71 (7.62)	29.29 (5.72)	33.41 (7.86)	5.35	0.0023
Education (years)	13.32 (4.61)	13.08 (2.58)	13.42 (1.98)	13.34 (2.47)	0.1453	0.9323

*There was a significant difference of age between the groups. SD, standard deviation. ^∗^degrees of freedom = 3.*

Education-level matched control subjects were recruited via an advertisement at Ruijin Hospital. Exclusion criteria included a lifetime occurrence of a DSM-IV-TR axis I disorder or neurological disease and the current use of any medication that affects cognitive functions.

All subjects provided written informed consent for participation and publication; the study was approved by the Ethics Committee of Ruijin Hospital affiliated with Shanghai Jiao Tong University School of Medicine in accordance with the Declaration of Helsinki. A portion of this patient sample were participants in a parallel study investigating the safety and effectiveness of capsulotomy in refractory OCD (clinical.trails.gov: NCT02375152).

### Neuropsychological and Clinical Assessment

We used the computerized version of the IGT to examine decision making under ambiguity (Bechara et al., 2000). The IGT involves ascertaining the profile of wins and losses associated with four card decks (A, B, C, and D). Cards can be grouped into two advantageous (C and D) and two disadvantageous (A and B) alternatives. The two disadvantageous decks (A and B) are considered ‘risky,’ as they yield large immediate wins, but intermittently result in even larger losses and ultimately lead to debt. The other two decks (C and D) deliver smaller wins with small intermittent losses, resulting in overall gain. Each trial involves the selection of one card from four card decks, with a total of 100 trials. To subjects, the outcome probabilities are initially unspecified, the total number of trials is unknown, and the objective is to win as much virtual money as possible. As the task progresses, the contingencies can be inferred based on the feedback from the previous outcomes. The test reflects decision-making ability under uncertain and ambiguous conditions that require the use of reward feedback. We adopted a cross sectional experimental design to eliminate the influence of learning effects on task performance, as reordering the decks between testing sessions based on reward properties is not sufficient to control for these effects if the basic rule underlying this particular task has been learned.

The three OCD patient groups were clinically evaluated using three psychiatric rating scales: The Yale-Brown Obsessive-Compulsive Scale (Y-BOCS) (Goodman et al., 1989), the Hamilton Depression Scale (HAMD) (Hamilton, 1986), and the Hamilton Anxiety Scale (HAMA) (Maier et al., 1988). Mean and standard deviation values for each group are shown in **Table 2**.

**Table 2:** Summary of the psychiatric rating scale scores for each patient group.

	Pre-operative	Post-operative short term	Post-operative long term	Welch ANOVA	
	Mean (*SD*)	Mean (*SD*)	Mean (*SD*)	*F^#^*	*P*
Duration of OCD in years	10.66 (7.12)	8.92 (3.55)	10.50 (4.85)	1.43	0.25
Y-BOCS	25.84 (7.10)	14.54 (1.89)	9.22 (1.54)	168.22	<0.001
HAMA	15.16 (7.41)	9.54 (2.25)	6.09 (1.75)	46.87	<0.001
HAMD	14.37 (6.67)	7.58 (1.93)	4.78 (1.52)	57.78	<0.001

*^#^degree of freedom = 2.*

### Surgical Procedure of Anterior Capsulotomy

Before the surgery, patients received MRI scans for determining the target coordinates (1.5 T; General Electric, Madison, WI, United States). During the surgery, a stereotaxic surgical frame (Elekta Inc, Stockholm, Sweden) was fixed to the skull under local anesthesia to ensure precise targeting. A localization box was temporarily fixed to the frame. Data were fed into a high-performance computer and the target was identified based on stereotactic MRI visualization of the internal capsule. Coordinates for the bilateral anterior capsule target were 22–25 mm anterior to the middle of the anterior commissure (AC) – posterior commissure (PC) line, horizontal to the AC - PC, and 18–20 mm lateral to the midline. Monopolar electrodes (Radionoics, Burlington, MA, United States) were subsequently led into the target brain points to a position previously detailed through a drilled skull hole. The position of the electrode was set by a micromanipulator placed on the frame. Neurological testing was carried out to ensure that there were no impairments of motor or sensory functions. Brain lesions were then made with radio-frequency-stimulation below and right of the target points, first on the right side, then on the left side. These radiofrequency lesions were created at 80°C for 60 s. Further details of the surgical procedure can be found in the previous reports (Liu et al., 2008; Zhan et al., 2014).

### Statistical Analysis

#### Patient Background and Psychiatric Rating Scales

Patients with OCD were divided into three groups according to their surgical status: (1) the pre-operative group, (2) the post-operative short-term group, and (3) the post-operative long-term group. Descriptive variables including age and education duration, together with clinical variables including illness duration and scores on clinical measures were first evaluated using the Brown–Forsythe variance test for equal variances. As the participant groups showed significant different variances in age [*F*(3,134) = 6.00, *p* = 0.0007], education duration [*F*(3,134) = 9.15, *p* = 0.000015], illness duration [*F*(2,104) = 3.92, *p* = 0.023], YBOCS [*F*(2,104) = 24.79, *p* = 0], HAMD [*F*(2,104) = 20.43, *p* = 0], and HAMA [*F*(2,104) = 27.35, *p* = 0], Welch tests were applied to the rest of the analyses for testing the differences in the mean. Rank-sum tests were then applied to compare the data in pairs.

#### IGT Performance

The task consisted of 100 trials, which were divided into five blocks of 20 trials for the purpose of analysis. For each block, the net score was calculated as the difference between the numbers of advantageous and disadvantageous choices. A mixed ANOVA was then applied to examine the net score differences within and between groups. To increase statistical power, we also calculated the net scores by combining the trials from the blocks 2 and 3, and the blocks 4 and 5 to further investigate the learning process in the *post hoc* analysis. Differences between groups were tested within these testing periods (Block 1, Block 2 + 3, and Block 4 + 5). Correlation analyses between the net scores and the descriptive/clinical variables were then carried out.

#### Strategy Analyses

A mixed between-within 4^∗^4 ANOVA was applied to analyze the main differences in the strategy and participant group factors. Strategy was the within-subject factor, which included the stay probabilities after: wins based on the choices from the good decks, losses based on the choices from the good decks, wins based on the choices from the bad decks, and losses based on the choices from the bad decks. Participant group was the between-group factor, which included the healthy control group, the pre-operation OCD group, the post-operation short-term OCD patient group and the post-operation long-term OCD patient group.

## Results

### Demographic Characteristics

We first evaluated the demographic characteristics of the participant groups; these are displayed in **Table 1**. No significant difference in education duration was found among the four groups. However, the HC group was significantly older than the patient groups, as the patient groups generally had lower education levels than HCs of the same age. *Post hoc* analyses showed that the HC group was older than the preO group (*z* = 2.63, *p* = 0.0086) and the postST group (*z* = 2.74, *p* = 0.0062), and the postST group was younger than the postLT group (*z* = -2.15, *p* = 0.032) (**Table 1**). It is unlikely that this age difference can explain the differences in task performance reported in the following section, as previous reports have indicated that there are no effects of gender, age, or education on IGT performance (Brand et al., 2006).

### IGT Performance

**FIGURE 1:**
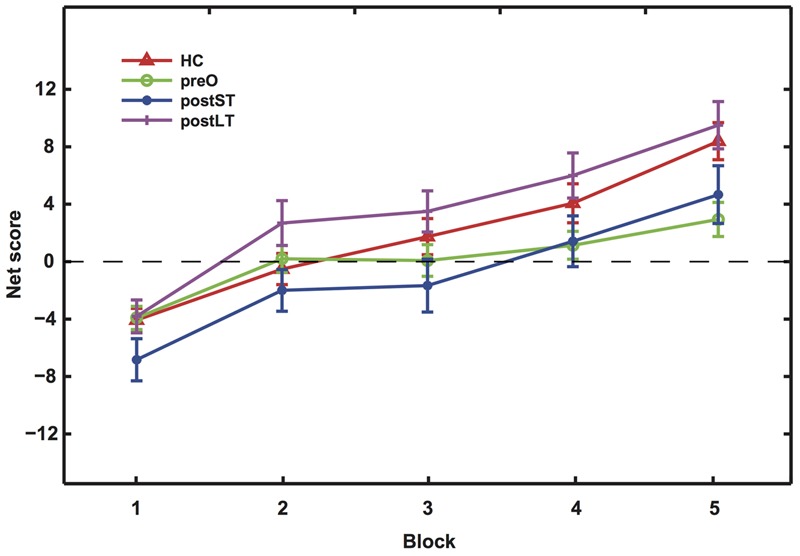
The net scores of the five blocks during the IGT. Mean net scores for each block were calculated as (C + D) – (A + B). The four curves represent the four participant groups as indicated by the legend labels. In Blocks 2 and 3, the postLT group’s scores were significantly higher than that of the postST group (*p* = 0.007); in Blocks 4 and 5, both the HC and postLT groups achieved significantly higher scores than the pre-operative group (*p*_HC_ = 0.0355; *p*_postLT_ = 0.0012).

The aim of the IGT task is to evaluate participants’ ability to learn to adjust their choice behavior based on reward feedback. The net scores showed learning effects in all four groups, as evidenced by their increase in each progressive block (**Figure 1**). In the IGT, cards from the bad decks initially provide large wins. We can infer from the results that subjects in all groups picked up on this feature, as they initially chose from the bad decks more frequently. Another feature of the IGT is that once the winning cards are drawn, the remaining cards in the bad decks are more likely to result in a large loss. The large, progressive increases in HCs net scores between blocks suggest fast learning of this reversal rule regarding win probability. Interestingly, the postLT group also demonstrated score increases consistent with fast learning, exhibiting no significant difference in the net scores from those of the HC group. As a result, the HC and the postLT groups completed the task with higher net scores than the other two groups. *Post hoc* analyses were conducted to examine these changes in more detail.

Scores and statistics are summarized in **Table 3**. Firstly, we found a significant difference in net scores between participant groups [ANOVA, *F*(3,134) = 6.54, *p* < 0.001]; and a significant difference between the blocks among groups, suggesting an overall learning effect [ANOVA, *F*(4,536) = 43.70, *p* = 0]. There were no significant interactions between participant groups and blocks [*F*(2,536) = 1.30, *p* = 0.21]. All groups performed similarly in the first block (*p* > 0.1), suggesting that the initial learning was similar in all groups. However, their performance started to differ in intermediate Blocks 2 and 3 [*F*(3,134) = 3.66, *p* = 0.0142]. A follow-up *t*-test *post hoc* analysis with Bonferroni correction indicated that the net score of the postLT group was significantly higher than the postST group (*p* < 0.01). The net scores of the participant groups remained significantly different in Blocks 4 and 5 [*F*(3,134) = 5.66, *p* < 0.01]. The follow-up *t*-test with Bonferroni correction *post hoc* analyses showed that the net scores of both the HC and the postLT group were significantly higher than that of the pre-operative group (p_HC_ < 0.05, p_postLT_ < 0.01).

**Table 3:** Net scores of each patient group in each testing block.

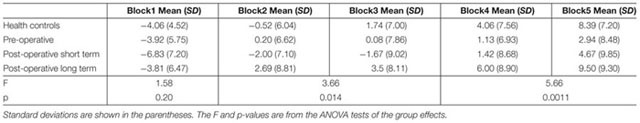

### IGT Choice Strategy

To further investigate participants’ choice behavior, we looked at how choice outcomes affected subjects’ strategy. We hypothesized that if the subjects had learned which two decks were good, they would tend to repeat their choices even after losing points from the good decks in a particular trial. On the other hand, if they lost points after picking cards from the bad decks, they would be more likely to switch to the good decks. In contrast, if the subjects did not know which decks were good, their switch/stay behavior would be more similar to a win-stay-lose-shift strategy.

**FIGURE 2:**
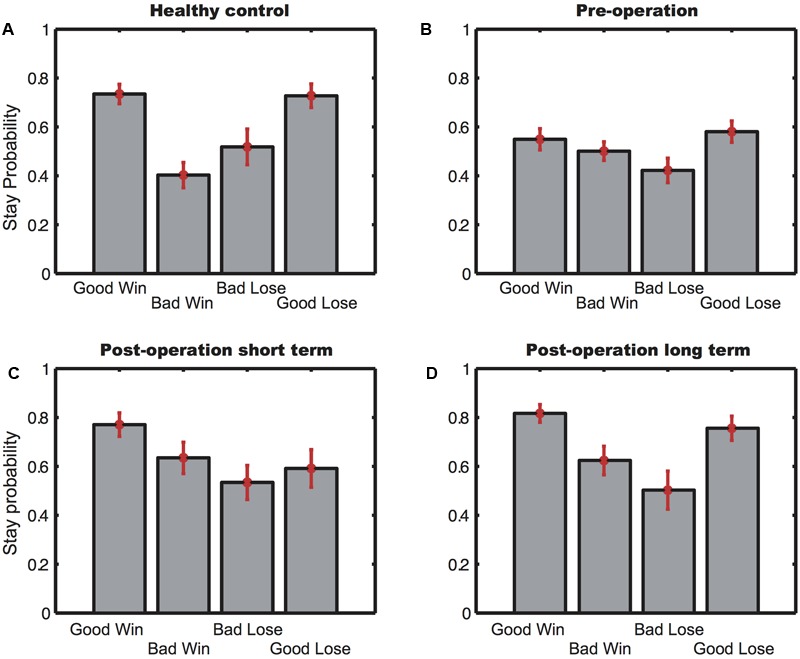
The stay probability of Block 5. **(A)** Stay probability for the healthy controls; **(B)** stay probability for the pre-operative group; **(C)** stay probability for the postST group; **(D)** stay probability for the postLT group. Error bars indicate SEM.

**Figure 2** shows the four participant groups’ tendency to repeat their choices, defined as stay probability, after wins and losses in Block 5. There was a main significant difference both among the trial conditions [*F*(3,288) = 4.73, *p* = 0.0030] and among the participant groups [*F*(3,96) = 3.43, *p* = 0.020] without significant interactions between the two. However, *post hoc* analyses revealed that subjects in both the HC group [*t*(28) = 4.41, *p* = 0.00] and the postLT group [*t*(25) = 3.028, *p* = 0.0057] tended to repeat their choices of the good decks regardless of whether they had just gained or lost points. In addition, their stay probability was significantly lower after they chose from the bad decks even if they had just won points. This choice pattern was not found in the subjects from the preO [*t*(46) = 1.79, *p* = 0.083] and the postST groups [*t*(20) = 0.88, *p* = 0.39]. Their choices were much less consistent, indicating that they did not understand which decks were advantageous and did not adopt an appropriate strategy, even in the final block of their test session.

### Clinical Measures

We evaluated subjects using three psychiatric rating scales commonly applied in the assessment of OCD patients. For the scores on all three scales, Welch ANOVA showed a main effect of operation [HAMA: *F*(2,104) = 46.87, *p* = 0.00; HAMD: *F*(2,104) = 57.78, *p* = 0.00); Y-BOCS: *F*(2,104) = 168.22, *p* = 0.00]. There was a trend of decreasing scores from the pre-operative to the postST and then to the postLT patients, suggesting the operation helped alleviate both the obsessive and compulsive behaviors in these patients.

While it might be tempting to associate the observed enhanced IGT performance with the improvements in OCD symptoms, we found that there was overall a lack of correlation between the patients’ psychiatric rating scale scores and their IGT performance, suggesting that the IGT and the psychiatric rating scales measured different dimensions of cognitive function. Only the HAMA scores of the postST patients showed a significant negative correlation (slope: -1.21) with their net scores (adjusted *R*^2^ = 0.096, *p* = 0.018).

## Discussion

The results of the present study converge with extensive research in the field of OCD demonstrating impaired decision making associated with the disorder, particularly in refractory OCD patients, and further suggest that AC results in improvement across obsessive-compulsive, depressive and anxious clinical symptoms as well as decision-making ability under ambiguity. Our findings indicate that improved decision making occurs over time as a result of the normalization of aberrant CSTC activity, rather than as an instantaneous result of the neurosurgical lesion, therefore possibly constituting further evidence for the role of the CSTC circuitry in decision-making ability. We found no evidence for an association between clinical symptoms and decision making, suggesting that different neural substrates are at play.

Anatomically, the prefrontal cortex (PFC) is essential for the neural processes that underlie decision making and its sub processes, including planning, inductive reasoning, reward processing and manipulating complex information (Cservenka and Nagel, 2012; Rebola et al., 2012). Studies often differentiate three sub-regions of the PFC: the dorsolateral prefrontal cortex (DLPFC), the orbitofrontal cortex (OFC) and the anterior cingulate cortex (ACC) (Fuster, 2001). The OFC occupies the ventral region of the PFC and can further be divided into the ventromedial and ventrolateral portions, with the ventromedial OFC most frequently implicated in OCD (Bechara et al., 1994). This area appears critical in integrating affective information relayed from the other limbic areas and directly signaling certain reward types. In environments where rewarding options may constantly change, the OFC plays an important role in adaptive behavior (Elliott et al., 2000). It maintains a representation of reward history and modifies former reward representations that may have become disadvantageous. Together with frequently reported neuroimaging findings of aberrant OFC activity in OCD patients, these lines of evidence lend support to an association between reward mechanisms and symptomatology of the disorder. OFC impairments are often acknowledged as the most supported account for the inadequate behavioral choices and inflexibility of behavioral change typically expressed in OCD, although the underlying circuitry is likely far more complex.

Interestingly, patients with lesions in the OFC show deficits in their IGT performance similar to OCD patients (Bechara et al., 1994). More generally, recent research has shown improvements in verbal memory, visual memory, visuospatial skills and executive function in treatment-refractory OCD following AC as compared to both a non-surgical treatment group and healthy controls (Gong et al., 2017). Our study therefore provides further evidence that abnormal OFC functionality may be a contributing factor of the OCD behavior. We postulate that AC may enhance OFC activity via its disinhibition, though the mechanism by which this occurs is not immediately obvious. One of the major pathways in the anterior internal capsule carries projections from the thalamus to the frontal cortex. However, these projections are excitatory. Corticopontine projections also pass through the internal capsule, although they are unlikely to affect the OFC. It is therefore likely that capsulotomy works by disrupting the basal ganglia’s internal connections between the caudate and lentiform nucleus. Modifying these connections may affect the output of the basal ganglia, leading to the disinhibition of the OFC. This indirect modulation could also explain why behavioral improvement is mostly observed only in the long run (Pepper et al., 2015).

We found that the AC improved OCD symptoms as indicated by clinical scores, which is consistent with previous reports (D’Astous et al., 2013). In addition, our observed outcome of improved decision-making ability occurring over time independently of symptom alleviation is in line with that of Csigo et al. (2010), and is indicative of possible differences in the neural substrates underlying clinical symptoms and neuropsychological difficulties in OCD. The objective of neurosurgical techniques is to influence the connections between cortical areas (e.g., orbitofrontal cortex and cingulum), the basal ganglia (particularly the caudate nucleus) and the medial dorsal thalamic nucleus. Our findings indicate that IGT performance is not directly influenced by the instantaneous changes caused by the disruption of these connections. The critical role of receptor function in the neural circuitry mediating behavioral control evokes the possibility that decision making as measured by the IGT task is modulated by dopamine or serotonin signaling. Neurochemical alterations of the serotonergic and dopamine systems are assumed to be involved in the mediation of OCD symptoms (Denys et al., 2004). Serotonin (5-HT) has received the most attention in OCD, as enhancement of 5-HT neurotransmission results in a therapeutic effect; potent 5-HT re-uptake inhibitors are effective treatments for OCD (Huang and Williams, 2007). However, there is evidence that dopamine receptor levels modulate impulsivity and effortful decision making in mice (Simon et al., 2013). Furthermore, striatal dopamine D2/D3-receptor availability was demonstrated to have an inverse relationship with response inhibition speed on the Stop-Signal Task (a measure of impulsive behavior) and a positive relationship with inhibition-related fMRI activation in the fronto-striatal neural circuitry in healthy individuals (Ghahremani et al., 2012). Further research is needed to determine whether long-term rebalancing of neurotransmitter levels after AC contributes to changes in decision-making ability and other cognitive facets, such as impulsivity, that are commonly impaired in OCD.

Various accounts consider neuropsychological impairment an intermediate phenomenon linking aberrant brain activity with clinical symptomatology (Zhang et al., 2015). Moreover, unaffected relatives of individuals with OCD consistently exhibit comparable deficits in decision making to probands (Viswanath et al., 2009; Cavedini et al., 2010). There is evidence that behavioral impairment in both is associated with gray matter abnormalities in regions including the orbitofrontal cortex, indicating a heritable component (Menzies et al., 2007). However, it remains unclear whether this component is a primary feature of disease circuitry or a compensatory mechanism for other primary features. Future studies aimed at improved characterization of the neural substrates of neuropsychological and clinical characteristics before and after surgical intervention can help to elucidate the complex overlap and divergence between these disease characteristics.

Finally, our sample was comprised of individuals with severe OCD that is resistant to conventional treatment methods. Studies have indicated that individuals with refractory OCD exhibit a higher degree of neuropsychological deficits than treatment responsive individuals in terms of attention, set-shifting ability, linguistic performance, decision making, impulsivity (Csigo et al., 2010). Interestingly, there is also evidence that poor performance on the IGT is predictive of poor pharmacological treatment outcomes (Cavedini et al., 2002). However, it is important to bear in mind that dysfunction within any of the other neural systems that feed into the OFC/vmPFC system can lead to similar decision-making impairments, particularly when the disorders are marked by recognized heterogeneity in neural and symptomatological manifestation.

## Conclusion

The current study corroborates previous findings supporting the effectiveness of AC for the treatment of refractory OCD. Our results revealed that long-term changes after AC in the brain circuit may be revealed by the patients’ improvements in IGT. These changes were not correlated to the psychiatric rating scales, which started to show improvements in the patients shortly after AC. Future studies will be needed to demonstrate the neural circuitry underlying both categories of change and how they are affected by AC across time.

## Author Contributions

TY, BS, and SZ conceived of the project. TY and YC designed and supervised the experiments. YC and TY analyzed the data. CZ, ST, KZ, HJ, HG, YX, TW, BS, and SZ collected the data. CZ, ST, TW, BS, and SZ performed the surgery. CZ, YC, KZ, and TY wrote the paper with inputs from all authors.

## Conflict of Interest Statement

The authors declare that the research was conducted in the absence of any commercial or financial relationships that could be construed as a potential conflict of interest.
